# *Eurotium cristatum* Fermented Loose Dark Tea Ameliorates Cigarette Smoke-Induced Lung Injury by MAPK Pathway and Enhances Hepatic Metabolic Detoxification by *PXR*/*AhR* Pathway in Mice

**DOI:** 10.1155/2021/6635080

**Published:** 2021-03-10

**Authors:** Xiang-Xiang Huang, Shuai Xu, Li-Jun Yu, Yu-Fei Zhou, Ying Zhou, Zhong-Hua Liu

**Affiliations:** ^1^Key Laboratory of Tea Science of Ministry of Education, National Research Center of Engineering Technology for Utilization of Functional Ingredients from Botanicals, College of Horticulture, Hunan Agricultural University, Changsha 410128, China; ^2^Hunan Provincial Key Laboratory for Germplasm Innovation and Utilization of Crop, Hunan Agricultural University, Changsha 410128, China

## Abstract

Cigarette smoke- (CS-) induced oxidative stress and inflammation in the lung are serious health problems. Primary and reprocessed tea products contain multiple antioxidants that have been reported to protect the lung against CS-induced injury. However, the beneficial effects of *Eurotium cristatum* fermented loose dark tea (ECT) and *Eurotium cristatum* particle metabolites (ECP) on CS-induced lung injury and its potential hepatic metabolic detoxification are still unclear. Therefore, sixty mice were randomly divided into six equal groups. CS-exposed mice were prevented or treated with ECP or ECT infusions for 12 or 8 weeks to determine the antioxidative stress, anti-inflammatory and potential metabolic detoxification of ECT and ECP. Thirty-six mice were randomly divided into six equal groups to observe the effects on hepatic metabolic detoxification by replacing daily drinking water with ECT. Results showed that CS significantly decreased the activities of glutathione peroxidase (GSH-Px) and superoxide dismutase (SOD) and upregulated the expressions of malondialdehyde (MDA), tumor necrosis factor alpha (TNF-*α*), interleukin-6 (IL-6), IL-8, and IL-1*β* in serum. These adverse effects were modulated by ECP and ECT. In addition, ECT upregulated the mRNA expression of pregnane X receptor (*PXR*) and cytochrome P450 (*CYP450*) in the liver on daily free drinking ECT mice group. Western blot analysis further revealed that in CS-exposed mice, ECP and ECT significantly decreased the phosphorylation of mitogen-activated protein kinase (MAPK) in the lung but upregulated the protein expressions of *PXR* and aryl hydrocarbon receptor (*AhR*) in the liver. Overall, our findings demonstrated that ECT and ECP protected against lung injury induced by CS via MAPK pathway and enhanced hepatic metabolic detoxification via *PXR* and *AhR* pathways. Therefore, daily intake of ECT and ECP can potentially protect against CS-induced oxidative and inflammatory injuries.

## 1. Introduction

Cigarette smoke (CS) contains more than 6,000 chemicals, and 40 of which are carcinogenic [1]. Exposure to CS triggers an increase in colossal free radicals and production of reactive oxygen species (ROS). Combined, they induce oxidative stress damages and lipid peroxidation and disrupt the oxidation/antioxidation system [2, 3]. In humans, the antioxidant system regulates accumulation of free radicals, which modulates oxidative damages. CS exposure disrupts antioxidative processes catalyzed by superoxide dismutase (SOD) and glutathione peroxidase (GSH-Px). At the same time, CS increases the expression of malondialdehyde (MDA), which is a product of lipid peroxidation that damages the lung [4]. In addition, oxidative stress promotes inflammation in the lung [5]. Moreover, the inflammatory responses from the initial occult exogenous oxidative stress are secondary sources of endogenous ROS. Overall ROS induces a vicious cycle of lung damage [6]. Smoking induces damages beyond the organs directly in contact with CS such as the liver. CS contains toxic chemical substances that increases oxidative stress, necroinflammation, and liver fibrosis [7]. In addition, smoking also disrupts the expression of important xenobiotic pregnane X receptor (*PXR*) and ligand-activated transcription factor aryl hydrocarbon receptor (*AhR*) in the liver. This in turn represses the expression of cytochrome P450 (*CYP450*), which adversely affects drug metabolism and detoxification in the liver [8, 9]. These studies underline the integral antioxidants play roles in preventing and reversing CS-induced lung and liver injury. Meanwhile, tea has been extensively demonstrated to be an excellent natural antioxidant [10]. *Eurotium cristatum* fermented loose dark tea (ECT) is a potential excellent antioxidant, attributed to the interaction between the tea and the fungi.

ECT is a type of primary dark tea (PDT) that fermented with *Eurotium cristatum* strains and covered with “golden flora.” Similar to Fu brick tea (FBT, a brick-shaped *Eurotium cristatum*-fermented dark tea), ECT is a daily beverage and nutritional supplement frequently consumed at the border and southern regions of China. Previous studies have shown that dark tea lowers lipid levels in the body [11] and participates in antiobesity [12], antioxidative [4], anti-inflammatory [13], and detoxification [14] processes. Further, FBT aqueous extract inhibits the mitogen-activated protein kinase (MAPK) and nuclear factor-erythroid 2-related factor-2 (Nrf2) signaling pathways in cells, thereby reducing oxidative stress levels [4]. ECT contains catechins, alkaloids, gallic acid (GA), and covers with lots of *Eurotium cristatum* particle metabolites (ECP). Research shows that catechins inhibit oxidative stress, lipid peroxidation, and the expression of proinflammatory mediators [15]. Caffeine (CAF) is the most abundant alkaloid in tea. It possesses antioxidative properties and protects against lung damages by modulation pulmonary inflammation [16]. Notably, GA inhibits oxidative stress and inflammation [17]. Several researches report that nonfungi-fermented teas including green and black tea modulate oxidative stress and undesirable inflammatory response caused by CS exposure [18, 19]. Yet, how the tea performs these functions, alone or in combination with ECP, remains to be validated.

This study therefore investigated the ameliorative effects of ECT and ECP on CS-induced oxidative stress and inflammation via MAPK pathway and hepatic metabolic detoxification via *PXR*/*AhR* pathway in mice. These findings provide a theoretical basis for the antioxidation, anti-inflammatory, and metabolic detoxification capabilities of ECT and ECP.

## 2. Materials and Methods

### 2.1. Animals

Ninety-six (experiment 1, *n* = 36; experiment 2, *n* = 60, respectively), 4-week-old SPF female C57BL/6 experimental mice were purchased from Changsha Slack Jingda Experimental Animal Co., Ltd. (Changsha, Hunan, China). They were reared under 12/12 hours of light and dark alternating cycles. The room temperature was maintained at 25 ± 1°C, under 40-70% relative humidity. Adequate food and water were provided throughout the experimental period. The protocol for the experiments was approved by the Animal Testing Committee of Hunan Agricultural University. Before the experiments, there was one-week adaptation period to the feeds.

### 2.2. Chemicals and Reagents

Raw PDT leaves, FBT, and Hua Juan Tea (HJT) were purchased from the Baishaxi Tea Factory Co., Ltd. (Yiyang, Hunan, China). The *Eurotium cristatum* strains were isolated from Baishaxi brick tea using the direct separation technique. Tea-lyophilized powder was prepared at the Hunan Agricultural University. Cigarettes were purchased from the Hunan China Tobacco Industry Co., Ltd. (Changsha, Hunan, China). Vacuum diaphragm pumps were purchased from Kamoer, KVP15-KL-1 (Shanghai, China). Analytical kits for GSH-Px, SOD, and MDA were purchased from Nanjing Jiancheng Bioengineering Institute (Nanjing, Jiangsu, China). ELISA kits for the analysis of tumor necrosis factor alpha (TNF-*α*), interleukin-6 (IL-6), IL-8, and IL-1*β* were purchased from Wuhan Hualianke Biotechnology Co. Ltd. (Wuhan, Hubei, China). Antibodies against p-p38 (4511S), p38 (8690S), p-Jun N-terminal kinase (p-JNK, (9251S)), JNK (9252S), p-extracellular-regulated kinase (p-ERK, (4370S)), and ERK (4695S) were purchased from Cell Signaling Technology, Inc. (Danvers, MA, USA). Monoclonal antibodies against *PXR* (ab192579), *AhR* (ab84833), and GAPDH (ab181602) were purchased from Abcam (Cambridge, UK). All other chemicals and reagents were at analytical grade.

### 2.3. Experiment 1. ECT Enhances Potential Metabolic Detoxification by Modulating PXR and CYP450 in Mice Liver

To evaluate the potential hepatic metabolic detoxification property of dark tea, 36 healthy mice were randomly divided into 6 groups (*n* = 6, per group): (1) control, (2) epigallocatechin gallate (EGCG), (3) PDT, (4) FBT, (5) HJT, and (6) ECT group. Notably, EGCG solution (80 mg/kg) and dark tea infusions (800 mg/kg) were used to instead of daily drinking water during the 8 weeks feeding period. Dark tea infusions were prepared using freeze-dried powder of PDT, FBT, HJT, ECT, and sterilized water. The relative mRNA expressions of *PXR*- and *CYP450*-related genes (CYP1A2, CYP2B1, CYP2C6, CYP3A1, CYP3A9, and CYP3A18) in mice liver tissues were assessed using real-time fluorescent quantitative PCR.

### 2.4. Experiment 2. ECT and ECP Ameliorate CS-Induced Lung Injury via MAPK and Enhance Hepatic Metabolic Detoxification via PXR and AhR in CS-Exposed Mice

To determine the antioxidative stress, anti-inflammatory, and potential metabolic detoxification properties of ECP and ECT on CS-induced damages, 60 mice were randomly divided into 6 groups (*n* = 10): (1) control, (2) CS model group, (3) CS exposure and ECP preventive group, (4) CS exposure and ECT preventive group, (5) CS exposure and ECP cure group and (6) CS exposure and ECT cure group. Preventive group, which received ECP/ECT for 12 weeks, was also exposed to air/CS for the entire experimental period. The interval between smoke exposure and feeding was about 5 hours per day. Cure group, which was exposed to air/CS for 8 weeks, received ECP/ECT from weeks 9 to 12. CS-exposed groups were exposed to 4% (vol/vol, CS/air) CS for 1 hour per day (12 cigarettes) for 12/8 weeks using a modified ventilated CS exposure chambers connected to 2 vacuum diaphragm pumps. One pump was connected to a burning cigarette to deliver fresh smoke (40 mL/min), whereas the other pump simultaneously delivered fresh air (960 mL/min) from outside the chamber. Control group were exposed to fresh air in a separate ventilated chamber using a similar procedure. Biochemical analyses were then performed to detect the effects of GSH-Px and SOD, as well as the expression of MDA, TNF-*α*, IL-6, IL-8, and IL-1*β* in mice serum. Western blot analysis was performed to detect the expression of phosphorylated p38/JNK/ERK MAPK and expression of *PXR*/*AhR* proteins.

### 2.5. Expression of PXR, CYP1A2, CYP2B1, CYP2C6, CYP3A1, CYP3A9, and CYP3A18 mRNA in the Liver

Total RNA of liver was extracted using cold TRIzol reagent (Tiangen, Beijing, China). The quality and concentration of total RNA at 260/280 nm was detected by spectrophotometry. cDNA was synthesized based on the FastQuant RT Kit (Tiangen, Beijing, China). The primers were synthesized at Sangon Biotech Co., Ltd. (Shanghai, China) and listed in Table 1. Amplification of the cDNA was performed using the SuperReal PreMix Plus kit (Tiangen, Beijing, China) following the manufacturer's instruction. The relative expression of *PXR*, CYP1A2, CYP2B1, CYP2C6, CYP3A1, CYP3A9, and CYP3A18 mRNA in the liver was then evaluated. The reaction was performed using the real-time PCR system (Roter-Gene Q), under the following parameters: predenaturation at 95°C for 10 minutes, subsequent denaturation at 95°C for 10 seconds, annealing at 55°C for 20 seconds, and elongation at 72°C for 30 seconds. The amplification was performed through 40 cycles, with the mRNA expression of the target gene analyzed based on the 2^−ΔΔCt^ equation normalized to the mean values of internal reference GAPDH gene.

### 2.6. Preparation of ECT

PDT leaves were sterilized at 121°C for 20 min, cooled, and thereafter inoculated with fungal suspension. The fermentation was conducted in an incubator at 28-30°C under 80% humidity. After drying at 90-120°C for 60 min, the ECT leaves were stored at -20°C refrigerator for further use.

### 2.7. Preparation of ECP and ECT Infusion

The gold ECP were first passed through a 100-mesh screen from ECT, and the ECP were extracted with boiling ultrapure water for 30 min in a boiling water bath. The ECP infusion was then filtered under reduced pressure. ECT were extracted using ultrapure boiling water for 30 min in a boiling water bath. The ECT infusion was then filtered under reduced pressure. ECP and ECT dosages depended on the weight of the mice (600 mg/kg). All samples were divided into small portions and stored under -20°C. The procedures were repeated weekly to keep the samples fresh.

### 2.8. HPLC Analysis

The concentration of catechins, alkaloids, and GA on PDT, ECT, and ECP infusions was analyzed using an Agilent LC-1260 high-performance liquid chromatography (HPLC) (Santa Clara, USA). The HPLC was equipped with a Welchrom C_18_ column (250 mm × 4.6 mm × 5 *μ*m). The mobile phase was ultrapure water (A) and N, N-dimethylformamide : methanol : glacial acetic acid = 39.5 : 2 : 1.5 (B) with a gradient elution of 9-14% B at 0-10 min, 14-23% B at 10-15 min, 23-36% B at 15-27 min, 36%-36% B at 27-31 min, 36%-9% B at 31-32 min, and 9% B at 32-37 min. The temperature of the chromatographic column was maintained at 30°C throughout all experiments. The injection volume was 10 *μ*L. The HPLC chromatogram was monitored at UV 278 nm.

### 2.9. Collection of Mice Serum, Lung Tissue, and Liver Tissue

Before sample collection, the mice were starved of food for 12 h. The mice were sacrificed following intraperitoneal anesthetization using pentobarbital sodium. Blood was drawn from the eyelids for serum collection. Briefly, after 1 h incubation at room temperature, the blood was centrifuged for 10 min 2500 r/min for 10 min under at 4°C. The upper serum was collected and stored at -80°C for future use. The lung and liver tissues were removed, washed 3 times with precooled saline, and dried. The right lung tissues were placed in formalin for preparation of pathological tissue sections. Some of the lung not in formalin and liver tissues were stored at -80°C.

### 2.10. Histological Evaluation

The right lung tissues were fixed in formalin for 3 days, embedded in paraffin, and thereafter stained using hematoxylin and eosin solution (H&E). The sections were observed under an optical microscope at 200x magnification to assess any morphological changes in lung tissue.

### 2.11. Biochemical Analysis

The oxidative stress index in mice serum was assessed by measuring the catalytic activities of GSH-Px and SOD, as well as the expression of MDA using ELISA kits (Nanjing Jiancheng, Nanjing, Jiangsu, China). On the other hand, the inflammatory responses were assessed by measuring the concentrations of TNF-*α*, IL-6, IL-8, and IL-1*β* using ELISA kits (Hualianke, Wuhan, Hubei. China). All ELISA kits were strictly used based on the manufacturer's instructions.

### 2.12. Western Blot Analysis

The MAPK proteins in the lung and *PXR*/*AhR* proteins in the liver were extracted using total protein extraction kit (Solarbio, Beijing, China), and quantified based on the BCA protein assay kit (Solarbio, Beijing, China). Thereafter, 20 *μ*g of denatured proteins were separated in 10% polyacrylamide gel using electrophoresis and then transferred to PVDF membrane (80 V, 30 min, 120 V, 60 min). The membranes were blocked for 1 h at room temperature using 5% nonfat milk in TBS-Tween (TBST), washed 3 times using TBST, and then incubated overnight at 4°C with several primary antibodies (p-p38 and p38, p-JNK and JNK, p-ERK and ERK, *PXR* and GAPDH, *AhR* and GAPDH (1 : 10000)). After three washes with TBST, the membranes were reincubated for 1.5 h at room temperature with horseradish peroxidase-conjugated secondary antibodies in TBST supplemented with 5% BSA. After 3 washes with TBST, the immunoblots were visualized after chemiluminescence. Protein concentrations were quantified by using Image J software.

### 2.13. Statistical Analysis

Data was analyzed using Prism version 7 (GraphPad Software, La Jolla, CA, USA). Difference between groups was analyzed using one-way ANOVA and Student's *t*-tests, based on Fisher's LSD. Continuous variables were expressed as mean ± SD. Statistical significance was set at *P* < 0.05 or *P* < 0.01.

## 3. Result

### 3.1. HPLC Analysis

**Figure 1:**
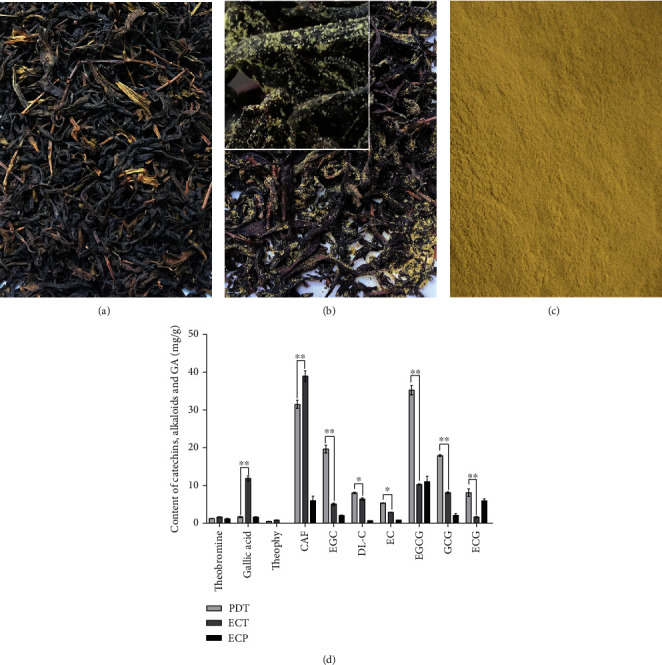
Photos of (a) PDT, (b) ECT, and (c) ECP. (d) The concentration of catechins, alkaloids, and GA in PDT, ECT, and ECP by HPLC method. ^∗^ and ^∗∗^ represent significant difference at *P* < 0.05 and *P* < 0.01 level, respectively.

Catechins, alkaloids, and GA were the major metabolites detected in PDT (Figure 1(a)), ECT (Figure 1(b)), and ECP (Figure 1(c)). As shown in Figure 1(d), after fermentation, total content of catechins in ECT was 34.37 ± 0.79 mg/g, decreasing by 63.54% in PDT (*P* < 0.01). However, the concentration of GA was 11.86 ± 0.49 mg/g with an increase percentage of 85.85 (*P* < 0.01). Three alkaloids (theobromine, theophy, CAF) all increased in ECT. Notably, CAF was the most abundant alkaloid at 38.99 ± 0.99 mg/g in ECT. All catechins, alkaloids, and GA were only slightly detected in ECP, perhaps due to the fact the detection method for ECP is different from the others. The decrease in ECT polyphenols was due to fermentation, in which the polyphenols were concerted to bioactive theabrownins [20]. These findings suggest that *Eurotium cristatum* fermentation changes the composition of metabolites on ECT compared to PDT, with the fungi retaining several bioactive and potential antioxidation substances.

### 3.2. Effects of EGCG, PDT, FBT, HJT, and ECT on the Expression Level of PXR- and CYP450-Related Genes (CYP1A2, CYP2B1, CYP2C6, CYP3A1, CYP3A9, and CYP3A18) in Liver Tissues

**Figure 2:**
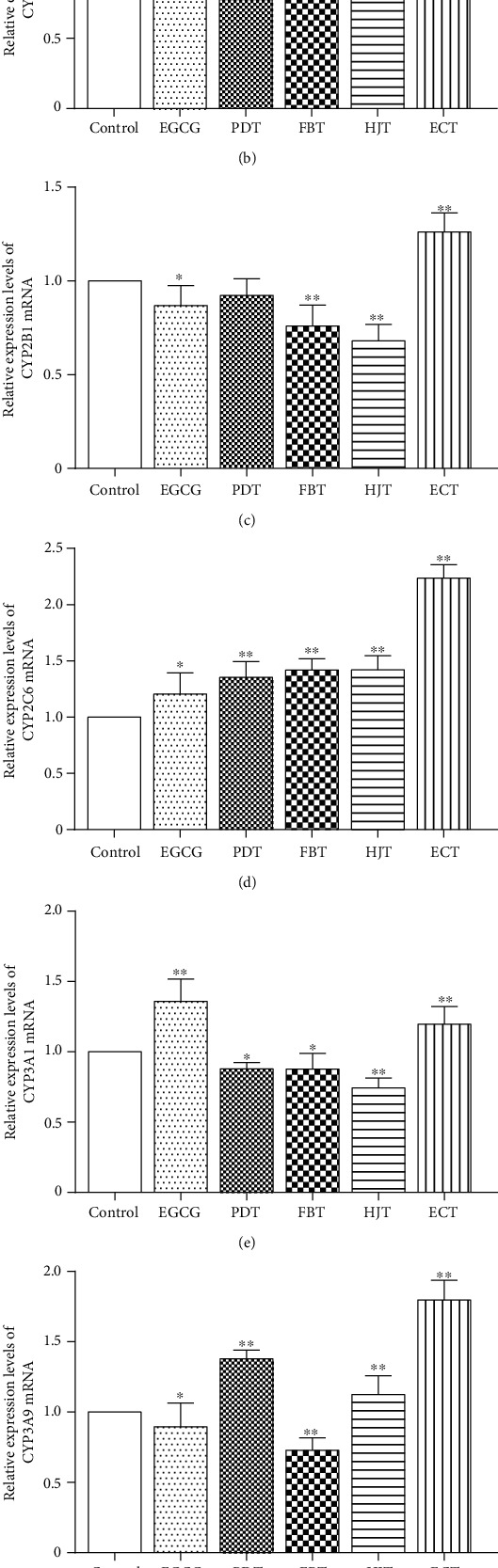
Effects of EGCG, PDT, FBT, HJT, and ECT on the expression of *PXR* and *CYP450* mRNA in mice liver. (a–g) Represent changes in *PXR*, CYP1A2, CYP2B1, CYP2C6, CYP3A1, CYP3A9, and CYP3A18, respectively. Values represents mean ± SD. ^∗^Statistical significance at *P* < 0.05; ^∗∗^statistical significance at *P* < 0.01.

As shown in Figure 2, compared with the control group, PDT and ECT significantly increased the expression of *PXR* mRNA. FBT and ECT induced a significant increase in the expression of CYP1A2 mRNA. ECT significantly increased the mRNA expression of CYP2B1. PDT, FBT, HJT, and ECT all significantly increased the mRNA expression of CYP2C6. EGCG and ECT significantly increased the mRNA expression of CYP3A1. PDT, HJT, and ECT significantly increased the expression of CYP3A9 mRNA. The expression of CYP3A18 mRNA decreased in all groups. These findings demonstrate that ECT plays a significant role in upregulating the expressions of *PXR*- and *CYP450*-related genes (CYP1A2, CYP2B1, CYP2C6, CYP3A1, CYP3A9, and CYP3A18). Also, the effect of ECT was superior to that of EGCG, PDT, FBT, and HJT. Statistical significance was set at *P* < 0.01 for all the analyses.

### 3.3. Histological Status of Mice Lung Tissues

Mice exposed to CS gradually but slowly gained weight with exposure frequency. Their fur turned yellow, and compared to controls, were rough and dull. Some mice shed their hair, lost appetite, and moved relatively slowly. After CS exposure, it took about 30 min for the mice to recover from the depressive symptoms.

**Figure 3:**
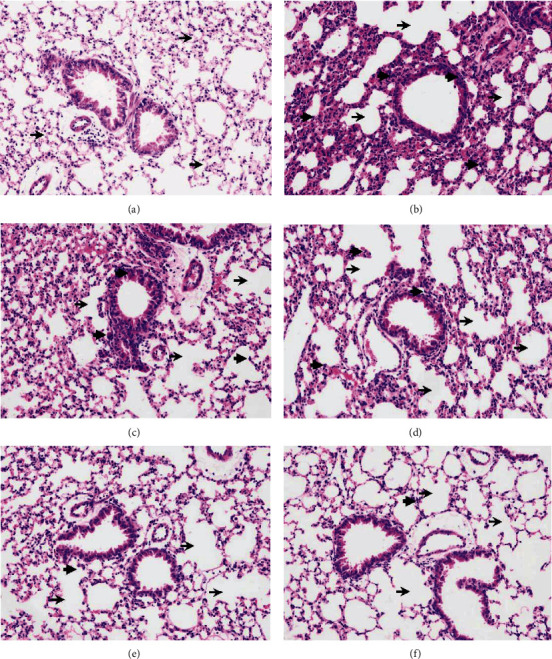
The effects of ECP and ECT on pathological appearance in the lung tissues exposed to CS (200x). (a) Control group shows normal lung tissue with uniform alveolar spaces (thin arrow). (b) CS model group shows dilated alveolar spaces (thin arrow), inflammatory cell infiltration (thick arrow), epithelial cells in the tracheal cavity are shed, and pulmonary interstitial congestion. (c) CS with ECP preventive group shows dilated alveolar spaces (thin arrow) and inflammatory cell infiltration (thick arrow). (d) CS with ECT preventive group shows dilated alveolar spaces (thin arrow) and inflammatory cell infiltration (thick arrow). (e) CS with ECP cure group shows dilated alveolar spaces (thin arrow), but less inflammatory cell infiltration (thick arrow). (f) CS with ECT cure group shows dilated alveolar spaces (thin arrow), but less inflammatory cell infiltration (thick arrow). Thin arrows show dilated alveolar spaces, whereas the thick one shows infiltration of inflammatory cells. Preventive group, included ECP/ECT intake from weeks 1 to 12 of mice exposed to CS through the same period. The interval between smoke exposure and feeding was about 5 hours per day. Cure group, mice were exposed to CS commenced from weeks 1 to 8, after which the mice received ECP/ECT from weeks 9 to 12.

As shown in Figure 3(a), the bronchi and alveolar of mice in the control group were intact with normal alveolar spaces. In contrast, those of CS group displayed dilated alveolar spaces, infiltration of inflammatory cells, shedding of epithelial cells in the tracheal cavity, and congestion in the pulmonary interstitial space (Figure 3(b)). Intriguingly, ECP and ECT treatment prevented or reversed the development of these pathological changes (Figures 3(c)–3(f)).

### 3.4. Effects of ECP and ECT on the Activities of Serum Antioxidant Enzymes

**Figure 4:**
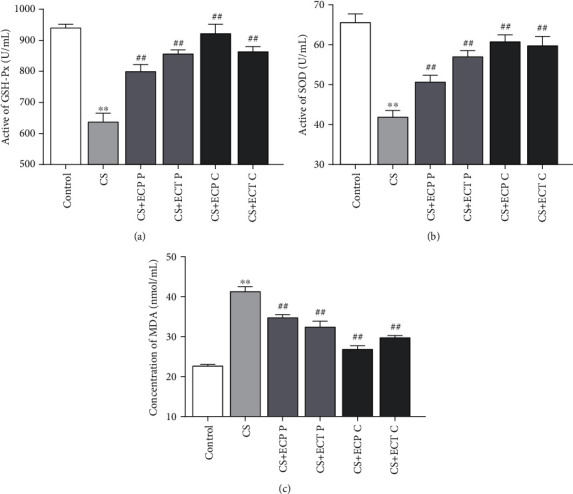
The effects of ECP and ECT on GSH-Px and SOD activities and the expression of MDA levels in serum of mice exposed to CS. Preventive group (P), included ECP/ECT intake from week s1 to 12 of mice exposed to CS through the same period. The interval between smoke exposure and feeding was about 5 hours per day. Cure group (C), mice were exposed to CS commenced from weeks 1 to 8, after which the mice received ECP/ECT from weeks 9 to 12. (a) Active unit of GSH-Px. (b) Activity of SOD. (c) Concentration of MDA. The values represent mean ± SD of each measure. ^∗∗^*P* < 0.01, compared with the normal control group; ^##^*P* < 0.01, compared with the CS model group.

The effect of ECP and ECT on the activity of antioxidant enzymes of mice was shown in Figures 4(a)–4(c). Compared with the control, CS exposure significantly decreased the activity of SOD and GSH-Px as well as upregulated the expression of MDA. In contrast, compared with control, ECP and ECT treatment significantly enhanced the activity of SOD and GSH-Px and significantly decreased the expression of MDA. The statistical significance was set at *P* < 0.01 for all the analyses.

### 3.5. Expressions of Inflammatory Cytokines in Serum

**Figure 5:**
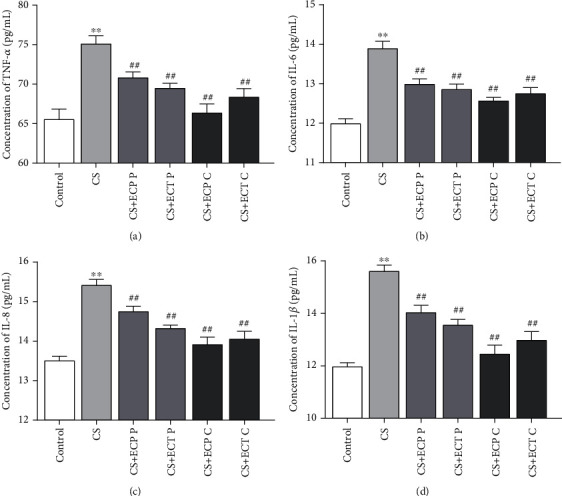
The effects of ECP and ECT on TNF-*α*, IL-6, IL-8, and IL-1*β* levels in serum of mice exposed to CS. (a–d) Concentration of TNF-*α*, IL-6, IL-8, and IL-1*β*, respectively. The measures represent mean ± SD. ^∗∗^*P* < 0.01, compared with the normal control group; ^##^*P* < 0.01, compared with the CS model group.

Compared with CS exposure significantly upregulated the expression levels of TNF-*α*, IL-6, IL-8, and IL-1*β* (Figures 5(a)–5(d)). However, ECP and ECT treatment conferred reduced expression levels of TNF-*α*, IL-6, IL-8, and IL-1*β* (*P* < 0.01) in CS-exposed mice.

### 3.6. Western Blot Analysis

**Figure 6:**
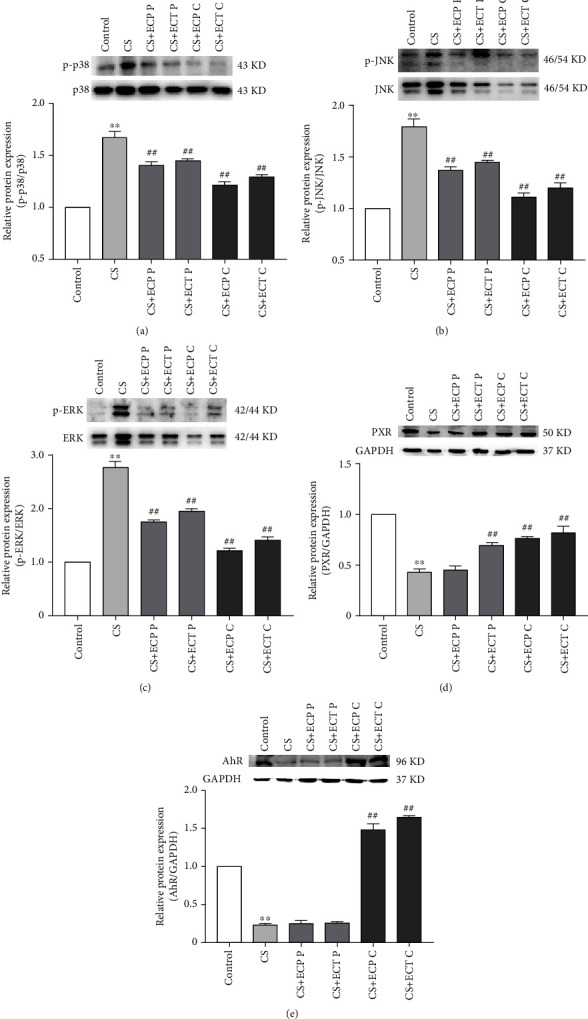
The effects of ECP and ECT on the expression of p38 and p-p38, JNK and p-JNK, and ERK and pERK proteins in the lung and *PXR* and *AhR* protein in the liver of mice exposed to CS. Western blot analysis for the expression of (a) phosphorylated p-p38 and total p38, (b) p-JNK and total JNK, (c) p-ERK and total ERK in lung tissues of mice exposed to CS. Expression of (d) *PXR* and (e) *AhR* proteins in the liver tissues of mice exposed to CS. Measurements represents mean ± SD. ^∗∗^*P* < 0.01, compared with the normal control group; ^##^*P* < 0.01, compared with the CS model group.

Phosphorylations of p38, JNK, and ERK proteinwere measured to uncover the molecular mechanism underlying modulation of CS-induced inflammatory response by ECP and ECT. Meanwhile, the expressions of *PXR* and *AhR* proteinwere evaluated to investigate the molecular mechanism underlying hepatic metabolic detoxification of ECP and ECT in CS-exposed mice. Compared with the control group, the phosphorylations of p38, JNK, and ERK proteinwere significantly (*P* < 0.01) upregulated in the lung of the CS mice (Figures 6(a)–6(c)). However, ECP and ECT intake, either as preventive or treatment intervention, regulated abnormal phosphorylations of p38, JNK, and ERK. In addition, compared with controls, CS exposure significantly (*P* < 0.01) repressed the expression levels of *PXR* and *AhR* protein (Figures 6(d) and 6(e)). However, preventive ECT or cure treatment with ECP and ECT significantly (*P* < 0.01) upregulated the expression of *PXR* protein (Figure 6(d)). Furthermore, cure treatment with ECP and ECT restored normal expression of *AhR* protein (Figure 6(e)).

## 4. Discussion

CS causes several health issues worldwide. Persistent CS exposure causes numerous chronic respiratory complications in the lung such as chronic obstructive pulmonary disease (COPD), emphysema, and in severe cases, lung cancer [21–23]. Also, smoking causes direct and indirect toxic effects on the liver, including oxidative stress, necroinflammation, and metabolic disorder [7]. The metabolic detoxification of the liver is related to the systemic antioxidant and anti-inflammatory effects. Studies have showed that several natural antioxidants including tea can modulate the oxidative stress, inflammation, and liver toxicity caused by CS [24–27]. Even though mechanism with which tea inhibits oxidative stress and inflammation has been described, how it mediates metabolic detoxification remains to be validated. Therefore, we first simulated daily tea intake, and in C57BL/6 mice, daily water intake was replaced with aqueous EGCG, PDT, FBT, HJT, and ECT infusions to evaluate the effect of different dark teas on the hepatic metabolic detoxification. We found ECT significantly increased the mRNA expression levels of *PXR* and *CYP450*. Yao et al. reported similar findings, in which tea was found to increase the expression of *CYP450*-related genes [28]. Subsequently, we established a lung injury mice model by CS to investigate the mechanism of ECT and ECP to ameliorate CS-induced lung injury and as well how it mediates hepatic metabolic detoxification.

ECT is an antioxidant with lower levels of total catechin but high alkaloid, GA, and theabrownins compared to PDT [29]. In this study, HPLC revealed that after fermentation, the concentration of alkaloids and GA in ECT increased, whereas that of total catechins decreased. However, only low levels of the metabolites were detected in ECP. Given that ECP is an *Eurotium cristatum* metabolite, the HPLC method for tea might not be suitable for measuring ECP. Elsewhere, Zou et al. showed that in addition to the 4 commonly known metabolites (echinulin, dehydroechinulin, neoechinulin A, and variecolorin O), cristatumin F, a novel metabolite, was also detected in *Eurotium cristatum* crude extracts isolated from Fu brick tea. Among them, cristatumin F exhibited scavenging effects on free radicals [30]. These findings demonstrated that ECT and ECP have high level of active antioxidant and anti-inflammatory substances. Based on these findings, we hypothesized that ECT and ECP could alleviate CS-induced lung injury by inhibiting oxidative damages and inflammatory responses and may also has the ability to enhance the hepatic metabolic detoxification.

CS exposure greatly impacts on the survival of mice. Compared with controls, mice in the ECP and ECT cure groups showed consistent unhealthy status during the first 2 months. These adverse events were significantly reversed after the cessation of smoking and the beginning of gavage during the final month. Also, ECP and ECT preventive groups significantly improved the adverse status of the mice. Pathological examination of the tissue sections revealed that the lung tissues of mice in the control group displayed normal structure with no inflammatory cell infiltration. In contrast, there were abnormal alterations in alveolar and epithelial cells and infiltration of inflammatory cells in CS model group. However, cure and preventive treatment of ECP and ECT significantly reversed these changes. The protective effects of ECP and ECT are associated with its antioxidative and anti-inflammatory effects.

Oxidative stress disrupts the antioxidant system and increases lipid peroxidation. Therefore, functions of SOD and GSH-Px antioxidant enzymes and MDA peroxidation are key indicators of oxidative stress in the body [4]. In this study, we found CS exposure significantly suppressed SOD and GSH-Px activities. Nevertheless, ECP and ECT treatment restored these changes. Notably, the effect was superior in cure groups than preventive groups. These findings underline the protective effect of ECP and ECT against CS-induced oxidative damages. Previous studies showed that CS-induced lung injury is associated with excess free radicals and lipid peroxidation [31]. Herein, the CS group exhibited higher MDA levels compared to the control group significantly. However, both preventive and cure groups with ECP and ECT significantly decreased CS-induced damages and expression of MDA. These findings suggest that ECP and ECT inhibit CS-induced oxidative stress.

To further clarify the pathophysiological effects of ECP and ECT on CS-exposed mice, we measured the concentration of 4 inflammatory markers including TNF-*α*, IL-6, IL-8, and IL-1*β*. In a separate study, it was found that TNF-*α* and IL-1*β* in human endothelial cells exposed to CS for longer periods were significantly higher than in nonsmokers [32]. Significant IL-1*β* increases in lung tissue of COPD patients and induces sputum production [33]. On the other hand, CS exposure significantly increased secretion of IL-8 in human bronchial epithelial cells [34]. IL-6 is a robust cytokine that activates proliferation of T and B cells and regulates inflammatory response. CS exposure increases infiltration of inflammatory cells and expression of IL-6 and TNF-*α* in bronchial alveolar lavage fluid (BALF) [35, 36]. In this study, compared to control group, CS exposure significantly upregulated the expression of TNF-*α*, IL-6, IL-8, and IL-1*β*. However, ECP and ECT treatment significantly downregulated the expression of the above proinflammatory cytokines. Notably, the effect of ECP and ECT was superior in cure groups than the preventive groups. This is probably because CS exposure in the cure groups was stopped after 8 weeks, whereas that of preventive groups continued for 4 more weeks, thus, the extended smoke exposure severely hindered recovery from the oxidative damages. Interestingly, ECP conferred better effects than ECT in the cure groups. In contrast, the effect of ECT in the preventive groups was superior to that of ECP. This tendency may be due to the fact that the mice in the cure groups were already injured before receiving ECP and ECT and that acute ingestion of CAF may exacerbate lung injury after ECT feeding [37]. Yet in the preventive groups, after long-term treatment with smoking and feeding, mice showed increased tolerance to the CAF in ECT. Therefore, in the preventive groups, ECT was more effective than ECP in ameliorating lung injury in mice as a result of its higher content of tea polyphenols and GA. Hence, we hypothesized that the use of mild antioxidants such as ECP in the early stages would be more effective in improving the oxidative stress and inflammatory response after cessation of long-term CS exposure. However, ECT consumption was more effective in improving oxidative stress and inflammatory responses during prolonged CS exposure.

MAPK signaling pathway regulates extracellular signaling in cells. The tertiary MAPK kinase pathway regulates important physiological and pathological processes including cell growth, differentiation, apoptosis, and inflammation. It further regulates expression of p38, JNK, and EKR proteins. ERK mediates cellular inflammatory and transcriptional activities. During COPD development, activated ERK promotes the release of proinflammatory cytokines such as TNF-*α*, IL-6, and IL-1*β*. This exacerbates inflammation in the airways and increases oxidative DNA and alveolar cell damages [38, 39]. p38 and JNK pathways are regulated by stress-induced signals and lung proinflammatory cytokines [40, 41]. Moreover, recent findings show that TNF-*α* activates the p38 MAPK signaling pathway, which induces asthma and the development of COPD [42]. CS metabolites induce phosphorylation of cellular H3S10 histones via the JNK and phosphatidylinositol 3-kinase/protein kinase B pathways, which directly promotes tumorigenesis [43]. In both COPD and non-COPD patients, CSE treatment upregulated the expression of IL-6 and IL-8 in lung bronchial cells and activated the p38 and JNK signaling pathways. Therefore, in general, CS induces proinflammatory responses that exacerbate COPD [44]. This study showed that CS exposure significantly upregulated the expression of phosphorylated p38, JNK, and ERK proteins and activated the MAPK signaling pathway. However, both preventive as well as ECP and ECT treatments reversed the above effects. Notably, the ECP and ECT protective effects were superior in cure than in preventive groups. This was in agreement with recent studies that showed that FBT reduces the level of UVB-induced oxidative stress in human keratinocytes by modulating the MAPKs/Nrf2 signaling pathway [45]. Also, it has been demonstrated that metabolites with antioxidant and anti-inflammatory properties such as GA, catechins, and *Eurotium cristatum* metabolites modulate the MAPK signaling pathway [4, 46, 47]. Therefore, our findings demonstrated that ECT and ECP inhibit CS-induced activation of lung MAPK signaling pathway, phosphorylation of p38, JNK, and ERK proteins, oxidative stress, and inflammation in mice.

*PXR*, a member of nuclear receptor superfamily NR1I2, plays a critical role in the metabolic detoxification system by detecting biological xenobiotics and triggering detoxification reactions, primarily expressed in the liver and intestine [48]. As already mentioned, *PXR* is a xenosensor that modulates the expression of xenobiotic-metabolizing enzymes and transporters. Therefore, this mediates the elimination of xenobiotics and endogenous toxic chemicals such as bile acids [49]. A recent study showed that exposing *PXR* knockout mice to 2,2′,4,4′,5,5′-hexachlorobiphenyl (PCB-153) markedly reduced the expression of GSH-Px and increased oxidative stress levels *in vivo*. On the other hand, PCB metabolites were significantly upregulated in mice liver, indicative of oxidative stress and DNA damage in the mice liver [50]. Naspinski et al. reported that *PXR* enhances cellular detoxification by upregulating expression of metabolizing enzyme, effectively protecting cells from benzopyrene- (BaP-) induced DNA damage [51]. These findings suggest that *PXR* protective against oxidative liver damage modulates expression of metabolic enzyme and enhances metabolic detoxification [49]. *AhR* is a xenobiotic receptor strongly expressed in the liver cells. It detects environmental toxins and regulates metabolism of xenobiotic [52]. Also, *AhR* participates in liver development, regulates liver regeneration, and inhibits tumor development. Moreover, mice models showed that *AhR* prevents activation of hepatic stellate cells and liver fibrogenesis [53, 54]. Numerous studies have also demonstrated that *AhR* alleviates oxidative stress, inflammation, and apoptosis induced by CS [55–57]. In this study, we found CS exposure significantly decreased the expression of *PXR* and *AhR*. However, both preventive and cure ECP as well as ECT increased the expression of *PXR* and *AhR* proteins after CS exposure. Therefore, findings of this study suggest ECP and ECT are potential antioxidants that could enhance hepatic metabolic detoxification.

**Figure 7:**
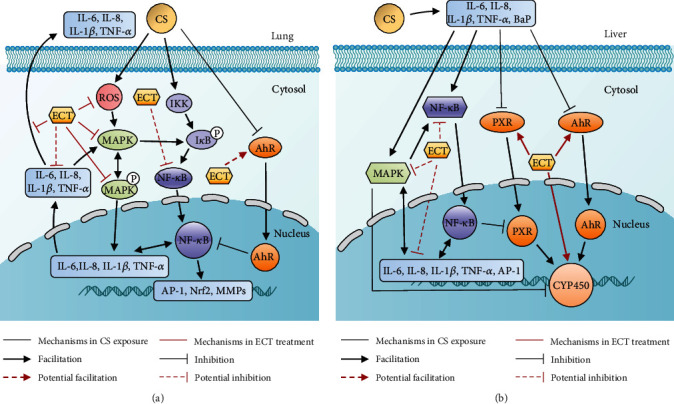
(a) Effect of ECT on MAPK, *AhR*, and NF-*κ*B signaling pathways in CS-induced lung injury in mice. CS upregulates the expression of MAPK and NF-*κ*B proteins but inhibits expression of *AhR* protein in the lung. It also induces secretion of proinflammatory cytokines. CS also induces oxidative stress and inflammation, causing lung injury. However, ECT ameliorated the lung injury. (b) Effect of ECT on MAPK, *PXR*, *AhR*, and NF-*κ*B signaling pathways in CS-induced liver injury in mice. CS activated the MAPK and NF-*κ*B signaling pathways, inhibited the expression of *PXR* and *AhR* proteins and the expression of *CYP450*-related genes, released proinflammatory cytokines, and disrupted metabolic detoxification processes in the liver. However, ECT may enhance the hepatic metabolic detoxification.

Meanwhile, increasing evidence suggests of a potential cross talk between MAPK, *PXR*, *AhR*, and other inflammatory signaling pathways. In particular, CS exposure activates the MAPK pathway in the lung [58], consistent with our findings. CS exposure also induces the overexpression of other inflammatory signaling pathways such as nuclear factor kappa B (NF-*κ*B) and TNF-*α* [59]. It also reduces the expression of *AhR* protein in lung, decreasing the protective capacity of *AhR* against inflammatory and oxidative damages in lung [60]. The effect of CS exposure on *PXR* in lung has not been reported, probably because *PXR* is mainly express in liver and intestine. Our findings demonstrated that ECT and associated ECP represses the MAPK signaling pathway and proinflammatory cytokine. Therefore, we speculate ECT and the fungi protects against CS-induced lung injury may be related to the *AhR*, MAPK, and NF-*κ*B pathways (Figure 7(a)). The effect of CS exposure on *PXR* and *AhR* signaling pathways in the liver is scarcely reported. However, mice models show that chronic CS exposure activates the MAPK and NF-*κ*B signaling pathways and induces the release of proinflammatory cytokines in the liver [59, 61]. Activated MAPK pathway inhibits expression of *CYP450*-related genes affecting drug metabolism and detoxification in the hepatocytes [62]. NF-*κ*B inhibits expression of *PXR* mRNA and disrupts the *PXR*-*CYP450* gene responses [48]. In this study, ECT and associated ECP enhanced the expression of *PXR*/*AhR* proteins in the liver induced by CS exposure. Hence, it is reasonable to hypothesize that CS exposure disrupts the *PXR*, *AhR*, MAPK, and NF-*κ*B signaling pathways and inhibit hepatic drug metabolism and detoxification. Moreover, ECT and associated ECP are antioxidant that can reverse these events (Figure 7(b)). However, we did not assess genes and proteins expression of MAPK and NF-*κ*B in the liver as well as NF-*κ*B, *PXR*, and *AhR* in the lung of the experimental mice. In addition, expression of key tissue molecular indicators for inflammation such as NF-*κ*B and activator protein-1 was not evaluated. Nevertheless, these researches will continue to be investigated in the future.

## 5. Conclusion

This study demonstrated that ECT and ECP significantly improved the health and restored normal pathophysiology in the lung induced by CS. Moreover, they remarkably enhanced the SOD and GSH-Px functions and downregulated the expression of MDA, IL-6, IL-8, IL-1*β*, and TNF-*α* in the serum. In addition, ECT and ECP downregulated phosphorylation of lung p38, JNK, and ERK proteins in mice. Furthermore, ECT remarkably upregulated the expression of mRNA for *PXR*- and *CYP450*-related genes. It also upregulated the protein expression of *PXR* and *AhR* in the liver. Overall, this study demonstrated that ECT and ECP protects against CS-induced oxidative stress and inflammatory damages in mice lung, via the MAPK signaling pathway. Meanwhile, ECT and ECP also protects against modulation of metabolic detoxification in the liver via the *PXR*/*AhR* signaling pathway. Accordingly, daily intake of ECT and ECP can potentially protect against CS-induced oxidative and inflammatory injury.

## Figures and Tables

**Table 1:** Primers used in the quantitative real-time polymerase chain reactions.

Gene	Gene accession number	Primer sequence 5′-3′	Product size (bp)
*PXR*	—	F: GACGGCAGCATCTGGAACTAC	112
		R: TGATGACGCCCTTGAACATG	
CYP1A2	NM_012541	F: AAGCGCCGGTGCATTG	1,882
		R: GCAGGAGGATGGCTAAGAAGAG	
CYP2B1	J00719	F: CCCCATGTCGCAGAGAAAGT	1,567
		R: GCGGTCATCAAGGGTTGGTA	
CYP2C6	BC100092	F: CTCTGTTGCTCCTGCTGAAGTG	1,268
		R: TGCCAACCACACGATCAATC	
CYP3A1	L24207	F: TTATGCTCTTCACCGTGATCCA	2,015
		R: GATCAATGCTGCCCTTGTTCTC	
CYP3A9	NM_147206	F: GGCCTACAGCATGGATGTGA	1,976
		R: CTGTGGGTTGTTAAGGGAATCAA	
CYP3A18	NM_145782	F: AAGCACCTCCATTTCCTTCATAAT	2,005
		R: TCTCATTCTGGAGTTTCTTTTGCA	
GAPDH	AF106860	F: CCTTCCGTGTTCCTACCC	2,039
		R: CCCAAGATGCCCTTCAGT	

F: forward; R: reverse.

## Data Availability

The data used to support the findings of this study are available from the corresponding author upon request.

## References

[1] Fowles J., Dybing E. (2003). Application of toxicological risk assessment principles to the chemical constituents of cigarette smoke. *Tobacco Control*.

[2] Pace E., Ferraro M., di Vincenzo S. (2014). Oxidative stress and innate immunity responses in cigarette smoke stimulated nasal epithelial cells. *Toxicology In Vitro*.

[3] Aboulmaouahib S., Madkour A., Kaarouch I. (2018). Impact of alcohol and cigarette smoking consumption in male fertility potential: looks at lipid peroxidation, enzymatic antioxidant activities and sperm DNA damage. *Andrologia*.

[4] Xu X., Huang H., Yin X., Fang H., Shen X. (2020). Effect of lentivirus-mediated CFTR overexpression on oxidative stress injury and inflammatory response in the lung tissue of COPD mouse model. *Bioscience Reports*.

[5] Wu Y.-L., Lin A.-H., Chen C.-H. (2014). Glucosamine attenuates cigarette smoke-induced lung inflammation by inhibiting ROS-sensitive inflammatory signaling. *Free Radical Biology and Medicine*.

[6] Pandey R., Singh M., Singhal U., Gupta K. B., Aggarwal S. K. (2013). Oxidative/nitrosative stress and the pathobiology of chronic obstructive pulmonary disease. *Journal of Clinical and Diagnostic Research*.

[7] El-Zayadi A. R. (2006). Heavy smoking and liver. *World Journal of Gastroenterology*.

[8] Zevin S., Benowitz N. L. (1999). Drug interactions with tobacco smoking. An update. *Clinical Pharmacokinetics*.

[9] Kumagai T., Suzuki H., Sasaki T. (2012). Polycyclic aromatic hydrocarbons activate CYP3A4 gene transcription through human pregnane X receptor. *Drug Metabolism and Pharmacokinetics*.

[10] Zhao C. N., Tang G. Y., Cao S. Y. (2019). Phenolic profiles and antioxidant activities of 30 tea infusions from green, black, oolong, white, yellow and dark teas. *Antioxidants*.

[11] Chen G., Wang M., Xie M. (2018). Evaluation of chemical property, cytotoxicity and antioxidant activity in vitro and in vivo of polysaccharides from Fuzhuan brick teas. *International Journal of Biological Macromolecules*.

[12] Chen G., Xie M., Dai Z. (2018). Kudingcha and Fuzhuan brick tea prevent obesity and modulate gut microbiota in high-fat diet fed mice. *Molecular Nutrition & Food Research*.

[13] Wang Y., Xu A., Liu P., Li Z. (2015). Effects of Fuzhuan brick-tea water extract on mice infected with E. coli O157:H7. *Nutrients*.

[14] Zheng Q., Li W., Zhang H., Gao X., Tan S. (2019). Optimizing synchronous extraction and antioxidant activity evaluation of polyphenols and polysaccharides from Ya'an Tibetan tea (Camellia sinensis). *Food Science & Nutrition*.

[15] Lakshmi S. P., Reddy A. T., Kodidhela L. D., Varadacharyulu N. C. (2020). Epigallocatechin gallate diminishes cigarette smoke-induced oxidative stress, lipid peroxidation, and inflammation in human bronchial epithelial cells. *Life Sciences*.

[16] Weichelt U., Cay R., Schmitz T. (2013). Prevention of hyperoxia-mediated pulmonary inflammation in neonatal rats by caffeine. *European Respiratory Journal*.

[17] Singla E., Dharwal V., Naura A. S. (2020). Gallic acid protects against the COPD-linked lung inflammation and emphysema in mice. *Inflammation Research*.

[18] Chan K. H., Chan S. C., Yeung S. C., Man R. Y. K., Ip M. S. M., Mak J. C. W. (2012). Inhibitory effect of Chinese green tea on cigarette smoke-induced up-regulation of airway neutrophil elastase and matrix metalloproteinase-12 via antioxidant activity. *Free Radical Research*.

[19] Banerjee S., Maity P., Mukherjee S. (2007). Black tea prevents cigarette smoke-induced apoptosis and lung damage. *Journal of Inflammation*.

[20] Wang Q., Gong J., Chisti Y., Sirisansaneeyakul S. (2015). Fungal isolates from a Pu-erh type tea fermentation and their ability to convert tea polyphenols to theabrownins. *Journal of Food Science*.

[21] Bodas M., Silverberg D., Walworth K., Brucia K., Vij N. (2017). Augmentation of S-nitrosoglutathione controls cigarette smoke-induced inflammatory–oxidative stress and chronic obstructive pulmonary disease-emphysema pathogenesis by restoring cystic fibrosis transmembrane conductance regulator function. *Antioxidants & Redox Signaling*.

[22] Barnes P. J. (2016). Inflammatory mechanisms in patients with chronic obstructive pulmonary disease. *Journal of Allergy and Clinical Immunology*.

[23] Hecht S. S. (2002). Cigarette smoking and lung cancer: chemical mechanisms and approaches to prevention. *Lancet Oncology*.

[24] Kaisar M. A., Prasad S., Cucullo L. (2015). Protecting the BBB endothelium against cigarette smoke-induced oxidative stress using popular antioxidants: are they really beneficial?. *Brain Research*.

[25] Fischer B., Voynow J., Ghio A. (2015). COPD: balancing oxidants and antioxidants. *International Journal of Chronic Obstructive Pulmonary Disease*.

[26] Li S., Tan H.-Y., Wang N. (2015). The role of oxidative stress and antioxidants in liver diseases. *International Journal of Molecular Sciences*.

[27] Chen L., Mo H., Zhao L. (2017). Therapeutic properties of green tea against environmental insults. *The Journal of Nutritional Biochemistry*.

[28] Yao H. T., Hsu Y. R., Lii C. K., Lin A. H., Chang K. H., Yang H. T. (2014). Effect of commercially available green and black tea beverages on drug-metabolizing enzymes and oxidative stress in Wistar rats. *Food and Chemical Toxicology*.

[29] Xiao Y., Zhong K., Bai J.-R., Wu Y.-P., Zhang J.-Q., Gao H. (2020). The biochemical characteristics of a novel fermented loose tea by *Eurotium cristatum* (MF800948) and its hypolipidemic activity in a zebrafish model. *Food Science and Technology*.

[30] Zou X., Li Y., Zhang X. (2014). A new prenylated indole diketopiperazine alkaloid from Eurotium cristatum. *Molecules*.

[31] Jian T., Chen J., Ding X. (2020). Flavonoids isolated from loquat (Eriobotrya japonica) leaves inhibit oxidative stress and inflammation induced by cigarette smoke in COPD mice: the role of TRPV1 signaling pathways. *Food Function*.

[32] Barbieri S. S., Zacchi E., Amadio P. (2011). Cytokines present in smokers' serum interact with smoke components to enhance endothelial dysfunction. *Cardiovascular Research*.

[33] Pauwels N. S., Bracke K. R., Dupont L. L. (2011). Role of IL-1 and the Nlrp3/caspase-1/IL-1 axis in cigarette smoke-induced pulmonary inflammation and COPD. *European Respiratory Journal*.

[34] Nadigel J., Audusseau S., Baglole C. J., Eidelman D. H., Hamid Q. (2013). IL-8 production in response to cigarette smoke is decreased in epithelial cells from COPD patients. *Pulmonary Pharmacology Therapeutics*.

[35] Liu H., Ren J., Chen H. (2014). Resveratrol protects against cigarette smoke-induced oxidative damage and pulmonary inflammation. *Journal of Biochemical and Molecular Toxicology*.

[36] Lee J.-W., Shin N.-R., Park J.-W. (2015). *Callicarpa japonica* Thunb. attenuates cigarette smoke-induced neutrophil inflammation and mucus secretion. *Journal of Ethnopharmacology*.

[37] Li J., Li G., Hu J.-L. (2011). Chronic or high dose acute caffeine treatment protects mice against oleic acid-induced acute lung injury via an adenosine A2A receptor-independent mechanism. *European Journal of Pharmacology*.

[38] Li C., Yan Y., Shi Q. (2017). Recuperating lung decoction attenuates inflammation and oxidation in cigarette smoke-induced COPD in rats via activation of ERK and Nrf2 pathways. *Cell Biochemistry and Function*.

[39] Tebay L. E., Robertson H., Durant S. T. (2015). Mechanisms of activation of the transcription factor Nrf2 by redox stressors, nutrient cues, and energy status and the pathways through which it attenuates degenerative disease. *Free Radical Biology and Medicine*.

[40] Jin M., Xue C. J., Wang Y. (2019). Protective effect of hydroxysafflor yellow a on inflammatory injury in chronic obstructive pulmonary disease rats. *Chinese Journal of Integrative Medicine*.

[41] Kuo W. H., Chen J. H., Lin H. H., Chen B. C., Hsu J. D., Wang C. J. (2005). Induction of apoptosis in the lung tissue from rats exposed to cigarette smoke involves p38/JNK MAPK pathway. *Chemico-Biological Interactions*.

[42] de Jong K., Vonk J. M., Imboden M. (2017). Genes and pathways underlying susceptibility to impaired lung function in the context of environmental tobacco smoke exposure. *Respiratory Research*.

[43] Ibuki Y., Toyooka T., Zhao X., Yoshida I. (2014). Cigarette sidestream smoke induces histone H3 phosphorylation via JNK and PI3K/Akt pathways, leading to the expression of proto-oncogenes. *Carcinogenesis*.

[44] Hulina-Tomašković A., Heijink I. H., Jonker M. R., Somborac-Bačura A., Rajković M. G., Rumora L. (2019). Pro-inflammatory effects of extracellular Hsp70 and cigarette smoke in primary airway epithelial cells from COPD patients. *Biochimie*.

[45] Zhao P., Alam M., Lee S.-H. (2019). Protection of UVB-induced photoaging by Fuzhuan-brick tea aqueous extract via MAPKs/Nrf2-mediated down-regulation of MMP-1. *Nutrients*.

[46] Tanaka M., Kishimoto Y., Sasaki M. (2018). *Terminalia bellirica* (Gaertn.) Roxb. Extract and Gallic Acid Attenuate LPS- Induced Inflammation and Oxidative Stress via MAPK/NF-*κ*B and Akt/AMPK/Nrf2 Pathways. *Oxidative Medicine and Cellular Longevity*.

[47] Liang Y., Ip M. S. M., Mak J. C. W. (2019). (-)-Epigallocatechin-3-gallate suppresses cigarette smoke-induced inflammation in human cardiomyocytes via ROS-mediated MAPK and NF-*κ*B pathways. *Phytomedicine*.

[48] Pavek P. (2016). Pregnane X receptor (PXR)-mediated gene repression and cross-talk of PXR with other nuclear receptors via coactivator interactions. *Frontiers in Pharmacology*.

[49] Oladimeji P. O., Chen T. (2017). PXR: more than just a master xenobiotic receptor. *Molecular Pharmacology*.

[50] Egusquiza R. J., Ambrosio M. E., Wang S. G. (2020). Evaluating the role of the steroid and xenobiotic receptor (SXR/PXR) in PCB-153 metabolism and protection against associated adverse effects during perinatal and chronic exposure in mice. *Environmental Health Perspectives*.

[51] Naspinski C., Gu X., Zhou G. D., Mertens-Talcott S. U., Donnelly K. C., Tian Y. (2008). Pregnane X receptor protects HepG2 cells from BaP-induced DNA damage. *Toxicological Sciences*.

[52] Rothhammer V., Quintana F. J. (2019). The aryl hydrocarbon receptor: an environmental sensor integrating immune responses in health and disease. *Nature Reviews Immunology*.

[53] Yan J., Tung H. C., Li S. (2019). Aryl hydrocarbon receptor signaling prevents activation of hepatic stellate cells and liver fibrogenesis in mice. *Gastroenterology*.

[54] Moreno-Marín N., Merino J. M., Alvarez-Barrientos A. (2018). Aryl hydrocarbon receptor promotes liver polyploidization and inhibits PI3K, ERK, and Wnt/*β*-catenin signaling. *iScience*.

[55] Baglole C. J., Maggirwar S. B., Gasiewicz T. A., Thatcher T. H., Phipps R. P., Sime P. J. (2008). The aryl hydrocarbon receptor attenuates tobacco smoke-induced cyclooxygenase-2 and prostaglandin production in lung fibroblasts through regulation of the NF-kappaB family member RelB. *The Journal of Biological Chemistry*.

[56] Thatcher T. H., Maggirwar S. B., Baglole C. J. (2007). Aryl hydrocarbon receptor-deficient mice develop heightened inflammatory responses to cigarette smoke and endotoxin associated with rapid loss of the nuclear factor-kappaB component RelB. *American Journal of Pathology*.

[57] Rico de Souza A., Zago M., Pollock S. J., Sime P. J., Phipps R. P., Baglole C. J. (2011). Genetic ablation of the aryl hydrocarbon receptor causes cigarette smoke-induced mitochondrial dysfunction and apoptosis. *The Journal of Biological Chemistry*.

[58] Foronjy R., D'Armiento J. (2006). The effect of cigarette smoke-derived oxidants on the inflammatory response of the lung. *Clinical and Applied Immunology Reviews*.

[59] Savari F., Badavi M., Rezaie A., Gharib-Naseri M. K., Mard S. A. (2019). Evaluation of the therapeutic potential effect of Fas receptor gene knockdown in experimental model of non-alcoholic steatohepatitis. *Free Radical Research*.

[60] de Souza A. R., Zago M., Eidelman D. H., Hamid Q., Baglole C. J. (2014). Aryl hydrocarbon receptor (AhR) attenuation of subchronic cigarette smoke-induced pulmonary neutrophilia is associated with retention of nuclear RelB and suppression of intercellular adhesion molecule-1 (ICAM-1). *Toxicological Sciences*.

[61] Xie C., Zhu J., Wang X. (2019). Tobacco smoke induced hepatic cancer stem cell-like properties through IL-33/p38 pathway. *Journal of Experimental & Clinical Cancer Research*.

[62] Tang H., Xu M., Shi F. (2018). Effects and mechanism of nano-copper exposure on hepatic cytochrome P450 enzymes in rats. *International Journal of Molecular Sciences*.

